# Hyperfunction of Muscarinic Receptor Maintains Long-Term Memory in 5-HT_4_ Receptor Knock-Out Mice

**DOI:** 10.1371/journal.pone.0009529

**Published:** 2010-03-04

**Authors:** Luis Segu, Marie-José Lecomte, Mathieu Wolff, Julie Santamaria, René Hen, Aline Dumuis, Sylvie Berrard, Joël Bockaert, Marie-Christine Buhot, Valérie Compan

**Affiliations:** 1 Centre de Neurosciences Intégratives et Cognitives, CNRS UMR5228, Bordeaux University, Talence, France; 2 Centre de Recherche de l'Institut du Cerveau et de la Moelle Epinière, CNRS UMR7225 INSERM UMR S975, Paris, France; 3 Center for Neurobiology and Behavior, Columbia University, New York, New York, United States of America; 4 Institut de Génomique Fonctionnelle, Neurobiology, CNRS UMR5203 INSERM U661 Montpellier I & II University, Nîmes University, Montpellier, France; L'université Pierre et Marie Curie, France

## Abstract

Patients suffering from dementia of Alzheimer's type express less serotonin 4 receptors (5-HTR_4_), but whether an absence of these receptors modifies learning and memory is unexplored. In the spatial version of the Morris water maze, we show that 5-HTR_4_ knock-out (KO) and wild-type (WT) mice performed similarly for spatial learning, short- and long-term retention. Since 5-HTR_4_ control mnesic abilities, we tested whether cholinergic system had circumvented the absence of 5-HTR_4_. Inactivating muscarinic receptor with scopolamine, at an ineffective dose (0.8 mg/kg) to alter memory in WT mice, decreased long-term but not short-term memory of 5-HTR_4_ KO mice. Other changes included decreases in the activity of choline acetyltransferase (ChAT), the required enzyme for acetylcholine synthesis, in the septum and the dorsal hippocampus in 5-HTR_4_ KO under baseline conditions. Training- and scopolamine-induced increase and decrease, respectively in ChAT activity in the septum in WT mice were not detected in the 5-HTR_4_ KO animals. Findings suggest that adaptive changes in cholinergic systems may circumvent the absence of 5-HTR_4_ to maintain long-term memory under baseline conditions. In contrast, despite adaptive mechanisms, the absence of 5-HTR_4_ aggravates scopolamine-induced memory impairments. The mechanisms whereby 5-HTR_4_ mediate a tonic influence on ChAT activity and muscarinic receptors remain to be determined.

## Introduction

Serotonin (5-hydroxytryptamine 5-HT) modulates learning and memory, as reported by several pharmacological studies aimed at define the specific involvement of 5-HT receptors in these processes [1]. The administration of 5-HT_2A/2C_ or 5-HT_4_ receptor (5-HTR_4_) agonists or 5-HT_1A_ or 5-HT_3_ and 5-HT_1B_ receptor antagonists prevents memory impairment and facilitates learning in situations involving a high cognitive demand [1].

Despite large efforts to find out how to treat memory and learning impairments, treatment remained either in part or in whole ineffective. Among the eighteen 5-HT receptors, the 5-HTR_4_ has been itemized as an attractive target [2]. Numerous studies argued that 5-HTR_4_ contribute to regulate learning and memory. The systemic injection of the 5-HTR_4_ partial agonist RS 67333 produced increases in place and object recognition following systemic injections in both young and old rats during the acquisition phase [3], [4]. Furthermore, injecting RS 67333 during the consolidation phase enhanced object and place recognition memory only in old rats [3]. In a similar behavioral paradigm, a weak dose of SL 650155, another 5-HTR_4_ partial agonist, improved memorization [5]. Activating 5-HTR_4_ could thus attenuate memory deficits that installed gradually over time during development. Using these behavioral paradigms, 5-HTR_4_ antagonists (GR 125487, SDZ 205557) induced no change by themselves, but blocked the effects of RS 67333 and SL 650155, respectively [3], [5]. SL 650155 has further been found to suppress cognitive deficits of old rats using the linear maze test, as well as scopolamine-induced deficits in Morris water maze performances [5]. Moreover, SL 650155 and the cholinesterase inhibitor rivastigmine have a synergistic effect in old rat performances in the object recognition test and in the linear maze [5]. This synergism is interesting in view of a co-treatment of patients suffering from Alzheimer's disease because reduce the dose of cholinesterase inhibitors may decrease their side effects. Similarly, galanthaminium, a new cholinesterase inhibitor combined to a partial 5-HTR_4_ agonist (RS 67333), enhances place and object recognition performances in young and old rats [6].

The hippocampus is a key cerebral center involved in learning and memory [7], [8], [9], [10]. In the hippocampus, electrophysiological studies have described that CA1 neurons [11], [12] [13] [14] and granule cells in the dentate gyrus express 5-HTR_4_ [15]. In the prefrontal cortex (PFC), 60% of glutamatergic pyramidal neurons, express 5-HTR_4_ as determined by electrophysiological responses [16] and single cell PCR [17]. Numerous studies indicate that activating 5-HTR_4_ increases the release of acetylcholine (ACh) in both the cerebral cortex [18] and the hippocampus [14].

In contrast, whether the absence of 5-HTR_4_ alters memory has not been explored yet. We thus tested the performances of the 5-HTR_4_ KO mice in different behavioral paradigms that are mainly targeted toward the analyses of spatial behavior and memory. The present studies clearly indicate that the genetic ablation of 5-HTR_4_ did not alter learning and memory capacities in mice. In contrast, the deleterious effect of scopolamine, a cholinergic antagonist, on long term memory, was enhanced in mice lacking 5-HTR_4_. Other changes included decreases in the activity of the ACh synthesis enzyme, choline acetyl transferase (ChAT), in the PFC and septum, but not in the dorsal hippocampus in the 5-HTR_4_ KO in baseline conditions. ChAT activity was then studied following training and scopolamine treatment in mice of both genotypes.

## Materials and Methods

### Animals

The subjects were male 129/SvTer wild type (WT) and homozygous 5-HTR_4_ KO mice (generation detailed by Compan et al., 2004 [19]), obtained from heterozygous breeding. Mice were housed individually in standard transparent laboratory cages (26×12×14 cm) in a temperature-controlled (22±1°C) colony room, adjacent to the experimental room. Mice were provided with food and water available *ad libitum* and maintained on a 12∶12 light/dark artificial cycle (lights on at 6:00 h). Mice were tested over the light phase between 10:00 and 17:00 h. At the beginning of the behavioral experiments the subjects (n = 16 in each genotype) were 4–5 months old. One week before the beginning of the experiments, mice were handled and weighed each day by the experimenter. The genotype of each mouse was systematically controlled before each experiment. We performed experiments with different groups of WT and KO mice in accordance with the *Guide for Care and Use of Laboratory Animals* established by the Centre National de la Recherche Scientifique. All experiments were carried out according to ethical committee guidelines (Comité régional d'éthique de Montpellier, project agreement n°C34-172-13; animal experiment authorization n° 21CAE011).

### Locomotor Activity

The protocol used was adapted from Malleret et al. (1999) [20]. Locomotor activity was assessed using activity cages, where the mouse can move inside a cylindrical corridor. Eight photobeam cells connected to a computer that records the activity defined as the number of revolutions per min made by the subject. The mice were tested for 60 min on a single test day. One measure was taken every 5 min.

### Spontaneous Alternation

The Y-maze consisted of three identical arms (34×10×18 cm) converging at the center of a triangular area. A symmetrical Y shape was thus formed (120°C of angular deviation form each other). The apparatus was placed on the floor of the experimental room and the behavior of the mice was recorded using a video camera hung above it. Mice were placed individually in the central area to explore the maze freely for 6 min. The sequence of arms visited was recorded. Global activity was evaluated using the number of visits into the different arms. Alternation (*vs.* repetition) was defined as a visit to one arm followed by a visit to another arm. The frequency of spontaneous alternation was calculated as the ratio of actual alternation/possible maximum alternations.

### Elevated Plus Maze

This test was used to assess anxiety-like behavior in WT and 5-HTR_4_ KO mice to determine possible interfering emotional factors that may modulate performance in learning and memory tasks [21]. As described [20], the plus maze was made of four, dark gray Plexiglas arms, two open arms (67×7 cm) and two closed arms (67×7×17 cm) that formed a cross shape with the two open arms opposite each other. The maze was set at 55 cm above the floor and dimly illuminated (20 lux). Photobeam cells (connected to a computer), placed at two different levels along length of each arm, allowed detection of the passage of the animal from the central platform (7×7 cm) to any arm, and from the middle of an arm to its extremity (and return). Mice were placed individually on the central platform, facing an open arm and were allowed to explore the apparatus for 8 min. The number of entries into the four arms is related to global activity. The level of anxiety-like behavior in mice was evaluated by the relative number of entries or time spent in the open *vs*. closed arms (open arms/open + enclosed arms).

### Spatial Learning in a Water Maze

The apparatus was a white circular swimming pool (diameter: 140 cm, walls: 40 cm high), which was located in a room with various distal cues. The pool was filled with water (depth: 30 cm) maintained at 20°C, which was made opaque by the addition of a nontoxic white paint. Inside the pool was a removable circular platform in plexiglas (diameter: 13 cm) positioned such that its top surface was positioned at 0.5 cm below the water surface. The platform served as a refuge from the water and was generally located in the center of an arbitrarily defined quadrant of the maze. Data were collected using a video camera fixed to the ceiling and connected to a videotracking system (Videotrack Viewpoint, Lyon, France) and to a video recorder, both located in an adjacent room that received the individual home cages of mice currently tested.

Each mouse received a pretraining session that consisted in placing the mouse on the platform where it had to stay at least 15 s, followed by a 30 s swimming period, and ended by several trials of climbing onto the platform until each subject was able to climb without help. This non spatial procedure was required to avoid confusion between procedural aspects of the task and subsequent spatial performance [22].

During the learning stages proper (training), each animal was subjected to a daily four-trial session. Before the first trial of the first session, only, the mouse was placed for 15 s on the platform. Each trial consisted of releasing the mouse into the water facing the outer edge of the pool at one of the quadrants (except the quadrant where the platform was located) and letting the animal swim to escape to the platform before 90 s had elapsed. A trial terminated when the animal reached the platform, where it remained for 15 s. Mice that failed to find the platform within this time limit were invited to follow the finger of the experimenter which indicated the location of the platform, and had to stay onto the platform for 15 s before being removed and placed back in their home cage for a 15 min inter-trial interval. The cages were placed beneath a heat lamp to reduce core temperature loss. The releasing point differed at each trial (for example east, west, south and east if the platform was in the north quadrant), and different sequences of releasing points were used from day to day. The mice run by squads of eight, i.e., they had their first trial successively, then their second, until the fourth and last one; WT and 5-HTR_4_ KO mice ran alternately. At different stages of learning, animals were generally given a probe trial, which consisted of letting the mouse swim in the pool for a fixed duration (60 s), while the platform was removed. The releasing point was in the quadrant opposite to that where the platform was previously located.

Animal movements were recorded using Videotrack to calculate parameters of the performance of mice: 1) escape latency, i.e. the time required to escape to the platform from the releasing point (in seconds, s), and 2) path length, i.e. the distance covered by the mouse until it reaches the platform (in centimeters, cm), a measure of accuracy, as described [20]. Mean swim speed (path length/latency, in centimeters/second, cm/s) was also calculated.

Firstly, sixteen naive WT and 5-HTR_4_ KO mice were tested (water maze: experiment 1). On the first day, following the procedural pre-training, each mouse performed a visually guided orientation session, i.e., a series of four trials for which the submerged platform had a visible white cylinder (diameter: 4 cm, height: 7 cm) on the top, and was placed in a new location from trial to trial. Spatial learning proper started the day after (day 2) with the hidden platform alone, placed in a fixed location, to evaluate spatial reference memory performances. It was composed of two main stages: (1) acquisition (9 days, days 2–10), with the escape platform located in the center of the north quadrant; (2) reversal (4 days, days 11–14), with the platform located in the center of the east quadrant. On days 5 (mid-time acquisition), 10 (end of acquisition), and 14 (end of reversal), the mouse was given a fifth trial, which was a probe trial. A single (probe) trial was also conducted on day 15 (i.e. 24 h after the end of reversal), and finally on day 20 (i.e. 6 days after the end of reversal).

Secondly, when they were eight months old, the same mice were re-evaluated in the same experiment, using the same material and methods in a new laboratory environment (water maze: experiment 2). No significant differences were observed between the results of the original learning and its replicate. We thus used the following procedure. The mice performed a four-day spatial learning and half mice of both genotypes received an injection of scopolamine, a muscarinic cholinergic antagonist (0.8 mg/kg, i.p., n = 8 WT-Sco and n = 8 KO-Sco) or saline (n = 8, WT-Sal and n = 8 KO-Sal) 20 min before the first daily trial. The order of testing was counterbalanced between genotype and treatment. On the fourth and last day of learning, mice were given a fifth trial, which was a probe trial. A single (probe) trial was also conducted on the day after (day 5, i.e. 24 h later), but the mice were not injected on that day.

### ChAT Assay

Animals of both genotypes were sacrificed in basal condition and after the various treatments used and the prefrontal cortex (PFC: 1.2 mm^3^), the septum (3.9 mm^3^) and dorsal hippocampus (1.2 mm^3^) were microdissected from 1 mm-thick sections at −20°C using a micropunch following the landmarks of the stereotaxic atlas (PFC: A +1.6 mm, septum: 0.90 mm, dorsal hippocampus: A −1.70 mm, from bregma, see [23]. ChAT activity was measured as described by Santamaria et al. (2009) [24] using [^3^H]acetylcoenzyme A (4.4 Ci/mmol; Amersham, Arlington Heights, IL) as a substrate. Each assay was done in duplicate. Proteins were determined by the method of Bradford (1976) using bovine serum albumin as standard. ChAT specific activity is expressed as *p*mol ACh/min/µg proteins.

### Statistical Analyses

#### Behavioral study

The data of 1) locomotor activity (activity cage), 2) spontaneous alternation (Y maze) and 3) anxiety (elevated plus maze), were statistically evaluated using ANOVAs (StatView 5.1, SAS Institute) with the genotype (WT, KO) as the between-subject factor and respectively; 1) the number of revolutions, 2) the number of visits to different arms and the ratio of alternations, 3) the number of entries into arms and entries in open/(open + enclosed arms) as within-subject factors. The data related to the water maze experiments used the genotype (WT, KO; experiment 1 and 2) and treatment (Sco, Sal; experiment 2) as the between-subject factors. Trial, day, zones were the main within-subject factors of the analyses of variance (using StatView 5.01, SAS Institute Inc.). When the effect of main factors was significant (p<0.05), the Scheffé *F* test (p<0.05) was used for post-hoc analyses of individual group comparisons.

#### For the visually-guided orientation task, performance was evaluated across trials

For the spatial reference memory learning, performance was evaluated over days (averaged over the four training trials). We analyzed separately the acquisition (days 2–10) and reversal (days 11–14) stages for experiment 1. For the analyses of probe trials, we used 1) the path length in the whole swimming pool, assessing swim speed (probe trials have a fixed duration), 2) the relative path length in the target quadrant/path length in the whole pool (TargetQ/TotQs), and 3) the relative path length in the target platform zone/path length in the four equivalent zones (TargetPF/TotPs). These ratios, applied to each experimental group, allowed to determine a chance level (.25) for visiting the target quadrant zone or the platform zone, and to evaluate statistically (using a Student's *t* - test) whether swimming in the target quadrant or platform represents a spatial selectivity, which is significantly different from chance level.

#### Biochemical studies

For these analyses, genotype and treatment were used as independent variables. Parameters from biochemistry were used as dependant variables. If significant effects of genotype or genotype x treatment interaction were found, the independent variables were split for a one-way ANOVA (genotype or treatment) analysis. For multiple comparisons, we used the Scheffé *F* test with a probability of 0.01 and 0.05 as a significant difference.

## Results

### Locomotor Activity

Locomotor activity decreased regularly over time (5 min blocks/60 min test) in both WT and 5-HTR_4_ KO mice (*F*_(11,330)_ = 142.78, p<0.0001). The ANOVA revealed no significant effect of genotype or genotype x time interaction, which indicates equivalent abilities of habituation in a new environment and equivalent basal level of locomotion. However, over the first 5 min of the test session, ANOVA revealed a genotype effect (*F*_(1,30)_ = 7.08, p = 0.01) based on a lower level of activity in KO compared to WT mice (not illustrated), as similarly reported [19]. Over the next following 55 min, the activity of 5-HTR_4_ KO and WT was identical.

### Spontaneous Alternation in the Y Maze

The number of visits in the arms was weaker in 5-HTR_4_ KO than WT animals (*F*_(1,30)_ = 8.07, p = 0.008), suggesting a decreased exploratory activity (not shown). However, the mutant mice exhibited equivalent levels of spontaneous alternation compared to WT mice (around 87%). No significant (F<1) genotype effect was found for this measure of basic working memory ability.

### Anxiety-Related Behavior

Anxiety-related behavior of 5-HTR_4_ KO mice was examined using the elevated plus maze. No differences between mice of both genotypes were detected in the global activity or the anxiety-related parameters (all Fs<1, not shown).

### Spatial Learning in a Water Maze (Experiment 1)

Over the visually-guided orientation task (day 1), mice learned to orient themselves towards a cued platform through four successive trials. No significant differences were observed between mice of both genotypes for either escape latency and path length (both Fs<1). The KO and WT mice did not further differ in swim speed [F<1]. A significant trial effect for the escape latency [F_(3,90)_ = 11.19, p<0.0001] and path length [F_(3,90)_ = 7.36, p = 0.0002] was associated with a decrease of these parameters across the four trials. The ability of 5-HTR_4_ KO and WT mice to acquire the cued version of the water maze task was then identical, suggesting that the sensori-motor capacities and motivation were not modified in the absence of 5-HTR_4_.

**Figure 1:**
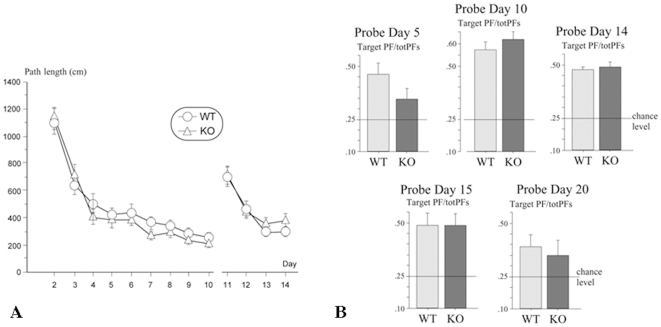
Spatial learning and reference memory of the 5-HTR_4_ knock-out mice (experiment 1). Left-hand side (A): Performance over days (2–14) for the acquisition (days 2–10) and the reversal (days 11–14) of spatial memory testing in the Morris water maze for wild-type (WT) and 5-HTR_4_ knock-out (KO) mice. The score on each day represents the mean ± s.e. of path length (cm). Right-hand side (B): Probe trials (60 s) on days 5, 10, 14, 15, and 20. Mean ± s.e. path length in the target platform zone/sum of path lengths in the four equivalent zones (target PF/totPFs) in WT and KO mice. Horizontal lines represent chance level.

Over days 2–10 (acquisition: spatial reference memory learning), the swim speed of the WT and KO mice was identical [F<1]. A progressive decrease in escape latency and path length over days was observed, indicating that mice of both genotypes learned the task. A significant day effect was found for the latency [F_(8,240)_ = 37.65, p<0.0001] and path length [F_(8,240)_ = 41.65, p<0.0001] (Fig. 1A, left-hand side). The 5-HTR_4_ KO and WT mice exhibited similar patterns of performance over time (interaction day x genotype [both Fs<1]). The swim speed of WT and KO mice was further decreased over time (day effect, *F*_(8, 240)_ = 29.78; p<0.0001). In summary, the performances of WT and KO mice did not differ in the water maze. The spatial reference memory is therefore not altered in the absence of 5-HTR_4_.

Over days 11–14 (reversal days: spatial reference memory learning), the displacement of the platform (see day 11, first day of reversal) increased the mean escape latency and path length in the same extent in both the WT and 5-HTR_4_ KO mice, which started at an identical level of performance (Fig. 1A). This deficit was compensated across subsequent days. Hence, mice lacking or not the 5-HTR_4_ acquired the new goal location over days 11–14 (escape latency: *F*_(3, 90)_ = 32.11, p<0.0001; path length: *F*_(3, 90)_ = 24.32, p<0.0001) with no significant differences between genotypes and no interaction effects (all Fs<1). For this period of time, swim speed was not significantly modified over days [F<1].

Over the probe trials (on days 5, 10, 14, 15 and 20), the absence of 5-HTR_4_ did not significantly affect swim speed. Globally, mice exhibited significant spatial selectivity (above chance level) when considering the target quadrant or the target platform zone (Fig. 1B). The spatial selectivity for the target quadrant did not differ between mice of both genotypes over probe trials (all Fs<1). 5-HTR_4_ KO mice exhibited significant spatial selectivity (i.e. above chance level), on all probe trials (day 5, t_(15)_ = 3.66, p = 0.002; day 10, t_(15)_ = 7.16, p<0.0001; day 14, t_(15)_ = 6.26, p<0.0001; day 15, t_(15)_ = 3.50, p = 0.003; day 20, t_(15)_ = 2.12, p = 0.05), as well as WT mice (day 5, t_(15)_ = 4.07, p = 0.001; day 10, t_(15)_ = 6.69, p<0.0001; day 14, t_(15)_ = 4.99, p = 0.0002; day 15, t_(15)_ = 3.82, p = 0.002; day 20, t_(15)_ = 2.80, p = 0.01). The analysis of spatial selectivity for the target platform did not reveal any significant effect of genotype for each probe trial (ps>0.05). The selective remembering of the target platform position was clearly exhibited, as for the quadrant zones, in WT mice (day 5, t_(15)_ = 4.35, p = 0.0006; day 10, t_(15)_ = 6.56, p<0.0001; day 14, t_(15)_ = 5.83, p<0.0001; day 15, t_(15)_ = 3.79, p = 0.002; day 20, t_(15)_ = 2.42, p = 0.03). It was different in 5-HTR_4_ KO mice, which showed a deficit in spatial retrieval shortly during the acquisition stage (day 5) and later when tested for long-term memory (day 20) (day 5, t_(15)_ = 1.60, p = 0.13; day 10, t_(15)_ = 8.81, p<0.0001; day 14, t_(15)_ = 5.11, p = 0.0001; day 15, t_(15)_ = 3.89, p = 0.001; day 20, t_(15)_ = 1.57, p = 0.14).

### Spatial Learning in a Water Maze Following Scopolamine (Experiment 2)

**Figure 2:**
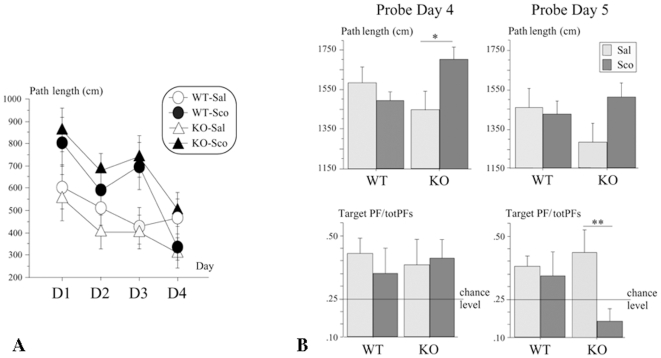
Spatial learning and reference memory of the 5-HTR_4_ knock-out mice (experiment 2). Effect of scopolamine injection (0.8 mg/kg, i.p., 20 min) on performance. Left-hand side (A): mean ± s.e. of path length (cm) over days (1–4) in wild type (WT) and 5-HTR_4_ knock-out (KO) mice. Right-hand side (B): Probe trials (60 s) on days 4 and 5. Top: Mean ± s.e. path length in the whole pool (swim speed) of the WT and KO mice. Bottom: Mean ± s.e. path length in the target platform zone/sum of path lengths in the four equivalent zones (target PF/totPFs) in WT and KO mice. Horizontal lines represent chance level. A significant treatment effect is noted (* p = 0.05, ** p = 0.02).

Over days 1–4 (acquisition), no significant genotype or treatment effect was observed for swim speed [Fs<1]. A progressive decrease in escape latency and path length across the four days was globally observed (Fig. 2A, left-hand side). ANOVAs revealed overall significant day effects for the latency [F_(3,84)_ = 5.32, p = 0.002] and path length [F_(3,84)_ = 8.98, p<0.0001]. The effect of treatment was significant [latency: F_(1,28)_ = 7.33, p = 0.01; path length: F_(1,28)_ = 4.86, p = 0.036], but not the effect of genotype, or the interaction treatment x genotype [all Fs<1]. Interestingly, the effect of treatment affected 5-HTR_4_ KO mice [latency: F_(1,14)_ = 6.10, p = 0.027; path length: F_(1,14)_ = 5.98, p = 0.028], not WT mice [latency: F_(1,14)_ = 1.87, p = 0.19; path length: F<1]. This suggests that 5-HTR_4_ KO mice reacted to the treatment at a dose, which did not significantly alter the performance of WT mice, at least over the acquisition of this familiar task. The performances on the last day of the learning test session (day 4) were no longer different between any treatments and genotypes (both Fs<1). The swim speed was, otherwise, characterized by a progressive decrease over days [F_(3,84)_ = 26.20, p<0.0001], in a similar extent in mice of both genotypes or following any treatment (both Fs<1).

On day 4 (probe trial), mice were tested immediately after the four daily training trials, i.e. remain under the influence of the treatment, but at that time the effect of the treatment on indices of performance was found ineffective (see supra). ANOVA conducted on swim speed revealed no significant effects of genotype and treatment, but a significant genotype x treatment interaction [F_(1,28)_ = 5.41, p = 0.03]. This was due to scopolamine-treated 5-HTR_4_ KO mice that exhibited increased swim speed [drug effect: F_(1,14)_ = 4.67, p = 0.05] while WT mice did not [F<1]; (Fig. 2B, right-hand side). Globally, mice exhibited weak spatial selectivity as considering the target quadrant, or the target platform zone. No significant genotype, treatment or, genotype x treatment interactions were observed (all ps>0.05). The selectivity for the target quadrant was exhibited by WT-Sal (t_(7)_ = 3.36, p = 0.01), but not by WT-Sco (t_(7)_ = 1.7, p = 0.13), KO-Sal (t_(7)_ = 1.4, p = 0.20), and KO-Sco (t_(7)_ = −0.4, p = 0.70). The same pattern of results was obtained considering spatial selectivity for the target platform zone, where it was significant in WT-Sal (t_(7)_ = 2.97, p = 0.02), but not in WT-Sco (t_(7)_ = 1.03, p = 0.34), KO-Sal (t_(7)_ = 1.37, p = 0.21), and KO-Sco (t_(7)_ = −2.04, p = 0.08).

On day 5 (probe trial), when tested 24 h later, without previous training or treatment, 5-HTR_4_ KO mice no longer exhibited increased in swim speed as related to their treatment on the day(s) before (Fig. 2B, right-hand side). ANOVA analyses of swim speed revealed no significant effects of genotype, treatment, and of the interaction genotype x treatment (all ps>0.05). Whatever their genotype and treatment, mice did not exhibit a significant spatial selectivity for the target quadrant (WT-Sal, t_(7)_ = −0.42, p = 0.68; WT-Sco, t_(7)_ = 0.20, p = 0.85; KO-Sal, t_(7)_ = 0.97, p = 0.36; KO-Sco, t_(7)_ = −1.74, p = 0.12). The pattern of results was different regarding selectivity toward the platform zone. ANOVA revealed no significant genotype effect (F<1), but a significant effect of treatment [F_(1,28)_ = 4.81, p = 0.04], which concerned 5-HTR_4_ KO [F_(1,14)_ = 7.32, p = 0.02], not of WT (F<1) mice. WT-Sal exhibited above chance-level preference above chance level (t_(7)_ = 3.30, p = 0.01), but not WT-Sco (t_(7)_ = 1.02, p = 0.34), KO-Sal (t_(7)_ = 2.06, p = 0.08), and KO-Sco (t_(7)_ = −1.93, p = 0.09).

In summary, results reveal that in a spatial version of the Morris water maze, learning, short- and long-term memories are not affected in the absence of 5-HTR_4_. In contrast, the deleterious effect of scopolamine on long-term memory performances was aggravated in the 5-HTR_4_ KO compared to WT animals. Since previous studies have reported that stimulating 5-HTR_4_ increases the levels of extracellular ACh in the frontal cortex [25], we set out, even if it was not the primary focus of our study, to test whether or not 5-HTR_4_ were required to maintain ChAT activity in selected brain areas under basal conditions and following the behavioral training tests.

### Adaptive Changes in ChAT Activity in 5-HTR_4_ KO Mice

**Figure 3:**
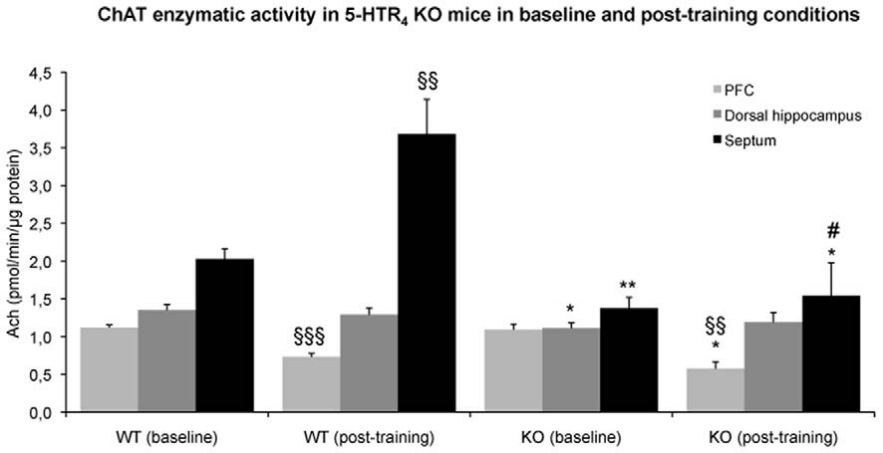
Reduced enzymatic activity of ChAT in the absence of 5-HTR_4_. Values are means ± s.e.m. of ChAT activity expressed in *p*mol ACh/min/µg protein for 7–8 WT and 6–7 KO mice in baseline conditions and 3–4 WT and 3–4 KO mice following the session of behavioral tests. A significant difference between genotypes or conditions is marked (* p<0.05, ** p<0.01 and §§ p<0.01, §§§ p<0.001, respectively). The genotype x condition interaction is significant (# p<0.05).

In baseline conditions, the enzymatic activity of ChAT was slightly decreased in the dorsal hippocampus (−18%, *F*_(1,12)_ = 5.46, p<0.05), and even more reduced in the septum (−33%, *F*_(1,12)_ = 11.24, p<0.01) but not in the PFC in 5-HTR_4_ KO compared to WT mice (*F*_(1,12)_ = 0.14, Fig. 3).

Following the behavioral tests (training session), the enzymatic activity of ChAT was markedly higher in the septum in WT mice compared to naive WT animals (+81%, *F*_(1,9)_ = 18.75, p<0.01; Fig. 3). No significant differences were detected in the septum between mutant mice under post-training and baseline conditions (interaction genotype x conditions: *F_1,16_* = 7.75; p<0.05). The enzymatic activity of ChAT was decreased in the PFC (−35%, *F*_(1,8)_ = 37.08, p<0.01), but unchanged in the dorsal hippocampus (*F*_(1,9)_ = 0.25) in trained WT compared to naive WT mice. Similar changes were also observed in both the PFC (−48%, *F*_(1,9)_ = 18.70, p<0.01) and in the dorsal hippocampus (*F*_(1,9)_ = 0.22) in trained 5-HTR_4_ KO compared to naive mutant mice (Fig. 3).

**Figure 4:**
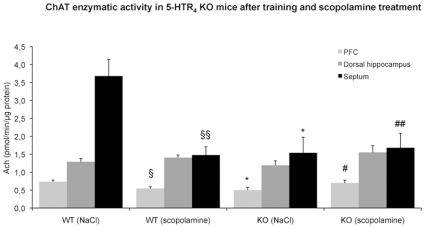
The effects of scopolamine on the enzymatic activity of ChAT were suppressed in the absence of 5-HTR_4_. Values are means ± s.e.m. of Ach levels expressed in pmol/min/µg protein for saline-treated mice (n = 3−4 WT, n = 3−4 KO) and for scopolamine-treated mice (n = 4−5 WT, n = 4−5 KO) following the behavioral test session. A significant difference between genotypes or treatments is noted (* p<0.05 and § p<0.05, §§ p<0.01, respectively). The genotype x treatment interaction is significant (# p<0.05, ## p<0.01).

The activity of ChAT was considerably decreased in the septum (−60%, *F*_(1,7)_ = 20.94, p<0.01) and in the PFC (−26%, *F*_(1,5)_ = 7.56, p<0.01) in scopolamine-treated and trained WT compared to saline-treated and trained WT mice (Fig. 4). These both changes were not observed in 5-HTR_4_ KO mice (septum: *F*_(1,5)_ = 0.05, PFC: PFC: *F*_(1,5)_ = 3.20 with significant genotype x treatment interaction, septum: *F*_(1,12)_ = 9.54, p<0.01; PFC: *F*_(1,10)_ = 8.84, p<0.05).

It was unchanged in the dorsal hippocampus in scopolamine-treated and trained WT compared to saline-treated and trained WT as well as KO mice (Fig. 4).

## Discussion

Findings clearly indicate that the null mutation of the mHtr4 gene did not induce learning and memory impairments. The memory performances of mutant mice were markedly decreased only following the administration of anticholinergic antagonist, scopolamine. It suggests that the muscarinic receptor function is enhanced in the absence of 5-HTR_4_, which has circumvented the loss-of-function mutation and maintained long-term memory performances, in baseline conditions. Unfortunately, the muscarinic function appears limited in its ability to adapt, reaching a “threshold limit”, because the deleterious effect of scopolamine on long-term memory was aggravated in the absence of 5-HTR_4_. The hyperfunction of muscarinic receptors may further contribute to compensate the decreased activity of ChAT detected in the 5-HTR_4_ KO mice, providing the additional first evidence that 5-HTR_4_ exert a tonic and positive control of the enzymatic activity of ChAT.

Over the last two decades, numerous studies have proposed that 5-HTR_4_ may contribute to learning and memory [26]. 5-HTR_4_ are located in cerebral structures, long known to influence memory, such as the hippocampus and the medial PFC [12], [14], [17], [27], [28]. In general, stimulating 5-HTR_4_ facilitates memory. For instance, injection of 5-HTR_4_ agonists enhances acquisition and performance of spatial learning [29], [30], [31], [32]. The 5-HTR_4_ antagonist decreases olfactory-associated memory [33]. In contrast, the present study indicates that learning and the spatial memory were not affected in 5-HTR_4_ KO mice. Using both the water and Y mazes, we further found that working (short-term) and long-term memory was unchanged in the absence of 5-HTR_4_. Results clearly suggest that learning and memory abilities are not altered when the mHtr4 gene encoding 5-HTR_4_ is disrupted, as far as the mutant mice are maintained in baseline conditions.

Results from pharmacological and present studies are not necessarily in discordance. It is often suggested that adaptive changes in neurons following the null mutation of any gene may occur over time during development, which may circumvent the loss-of-function mutation [34]. We have recently described that 5-HTR_4_ KO mice displayed a number of adaptive changes in serotoninergic neurons of the raphe nuclei [35]. In contrast, no major change was detected in 5-HT territories of projection, with however few exceptions [35].

These include a decreased density of 5-HTR_1A_ sites in the dorsal hippocampus (CA1) and in the septum in 5-HTR_4_ KO mice, in baseline condition [35]. Such reductions may likely contribute to maintain the level of performance of 5-HTR_4_ KO mice because 5-HTR_1A_ exerts an opposite effect on memory components, as reviewed [1]. Stimulating 5-HTR_1A_ impairs memory [36], [37], [38], [39], [40], [41], [42]. For instance, injecting 5-HTR_1A_ agonist, in the dorsal hippocampus, alters spatial and working memory [36], [38]. Recent work by Topic and colleagues [43] have further demonstrated that increased 5-HTR_1A_ binding in the hippocampus is associated with a decline in spatial memory of rats. Likewise, a transient overexpression of 5-HTR_1A_ during embryonic and perinatal development has detrimental effects on water-maze performance at adult stages [44]. In 5-HTR_1A_ KO mice the fear response to contextual clues increases [45]. Due to decreased 5-HTR_1A_ binding in their dorsal hippocampus, responses to learned fear in 5-HTR_4_ KO mice should be interesting to explore.

The present study indicates that cholinergic system function further adapts in the absence of 5-HTR_4_. The chronic blockade of muscarinic receptors revealed impairments in the long-term retention in 5-HTR_4_ KO mice for an ineffective dose of scopolamine in WT animals. Results accord those of Lelong and colleagues indicating that the injection of the BIMU1 and the 5-HTR_4_ partial agonist (RS 67333) prevented the deleterious mnemonic effects of scopolamine, using the Y-maze [32]. Likewise, stimulating 5-HTR_4_ with the intracerebroventricular injection of SC 53116 reduced the negative effects of scopolamine in the passive avoidance task [14].

Previous studies indicate that stimulating 5-HTR_4_ increases ACh release in both the frontal cortex and the hippocampus [14], [25], [46]. The present study provides a new evidence that 5-HTR_4_ mediates a tonic and excitatory influence on the enzymatic activity of ChAT in both the dorsal hippocampus and septum, but not in the PFC, in baseline conditions. It raises the question of the origin of this influence on cholinergic transmission. The regional and cellular distribution of 5-HTR_4_ has been extensively described in the brain of rodent, using selective radioligands [47], [48], combined with lesion or molecular studies [19], [49], [50]. Autoradiographic studies have shown high densities of 5-HTR_4_ in the rat hippocampus, using both the radiolabeled antagonists [^3^H]GR113808 [47] or [^125^I]SB207710 [48]. In the hippocampus, 5-HTR_4_ appear localized on both the somatodendritic and axonal zones of neurons from dentate granule cells to field CA3 and mainly on the soma of CA1 pyramidal cells [48]. Whether or not cholinergic neurons may express 5-HTR_4_ remained unknown. Beyond, results are in accordance with the ability of prucalopride (a 5-HTR_4_ agonist) combined with donepezil (an acetylcholinesterase inhibitor) to suppress scopolamine-induced amnesia [51].

The present study evidences that the genetic ablation of 5-HTR_4_ did not impair learning and memory under baseline conditions because of an adapted cholinergic hyperfunction, which however cannot totally overcome the absence of 5-HTR_4_.
